# Porcine deltacoronavirus resists antibody neutralization through cell-to-cell transmission

**DOI:** 10.1080/22221751.2023.2207688

**Published:** 2023-05-17

**Authors:** Sijin Xia, Wenwen Xiao, Xuerui Zhu, Shusen Liao, Jiahui Guo, Junwei Zhou, Shaobo Xiao, Puxian Fang, Liurong Fang

**Affiliations:** a State Key Laboratory of Agricultural Microbiology, College of Veterinary Medicine, Huazhong Agricultural University, Wuhan, People’s Republic of China; b The Key Laboratory of Preventive Veterinary Medicine in Hubei Province, Cooperative Innovation Center for Sustainable Pig Production, Wuhan, People’s Republic of China

**Keywords:** Porcine deltacoronavirus, cell-to-cell transmission, cell–cell fusion, neutralization, porcine aminopeptidase N

## Abstract

Porcine deltacoronavirus (PDCoV) is an emerging enteric coronavirus that has been reported to infect a variety of animals and even humans. Cell–cell fusion has been identified as an alternative pathway for the cell-to-cell transmission of certain viruses, but the ability of PDCoV to exploit this transmission model, and the relevant mechanisms, have not been fully elucidated. Herein, we provide evidence that cell-to-cell transmission is the main mechanism supporting PDCoV spread in cell culture and that this efficient spread model is mediated by spike glycoprotein-driven cell–cell fusion. We found that PDCoV efficiently spread to non-susceptible cells via cell-to-cell transmission, and demonstrated that functional receptor porcine aminopeptidase N and cathepsins in endosomes are involved in the cell-to-cell transmission of PDCoV. Most importantly, compared with non-cell-to-cell infection, the cell-to-cell transmission of PDCoV was resistant to neutralizing antibodies and immune sera that potently neutralized free viruses. Taken together, our study revealed key characteristics of the cell-to-cell transmission of PDCoV and provided new insights into the mechanism of PDCoV infection.

## Introduction

Porcine deltacoronavirus (PDCoV), an emerging enteropathogenic virus that belongs to the newly identified *Deltacoronavirus* genus in the family *Coronaviridae* [1], was first detected in Hong Kong in 2012 and has since spread rapidly to many countries worldwide [2–8]. Infection with PDCoV causes typical clinical symptoms, including acute diarrhoea, dehydration, vomiting, and even death in nursing piglets [9, 10]. Accumulating evidence shows that PDCoV also infects cattle [11], chickens [12], mice [13], and even humans [14], suggesting that PDCoV possesses cross-species transmission and zoonotic potential. It was recently proposed that PDCoV should be considered the eighth coronavirus to infect humans [15], highlighting the potential threat to human and animal health. A better understanding of PDCoV transmission should facilitate the development of efficient therapeutic approaches to fighting this emerging virus.

The coronavirus (CoV) spike (S) protein plays a crucial role in the viral invasion of host cells and is a major determinant of the tropism and pathogenesis of CoVs [16]. Similar to influenza virus hemagglutinin protein HA, human immunodeficiency virus (HIV) envelope glycoprotein Env, and other class I viral fusion proteins, CoV S is synthesized as a precursor protein that is subsequently cleaved into its receptor binding (S1) and fusion (S2) subunits [17, 18]. Previous research found that PDCoV S is a trimeric protein containing three receptor-binding S1 subunits and membrane fusion S2 subunits [19]. Importantly, PDCoV S is cleaved by cathepsins in the endosome and trypsin at the cell surface, which facilitates viral membrane fusion and host cell entry [20]. The detailed molecular mechanisms underlying PDCoV S-mediated infections need more extensive study to facilitate the future control of this virus.

For many enveloped viruses, infection is spread via two distinct modes: by diffusing through the extracellular environment and infecting new host cells or by propagating to neighbouring cells at sites of direct cell–cell contact [21–23]. The latter mode, called cell-to-cell transmission, gives the pathogen the ability to evade specific neutralizing antibodies and other intrinsic/innate antiviral responses, and thus promotes efficient viral infection and pathogenesis [24–27]. Evidence shows that cell-to-cell transmission is used in propagation or transmission to non-susceptible cells by many viruses, such as HIV, Epstein–Barr virus, and severe acute respiratory syndrome coronavirus 2 (SARS-CoV-2) [26–29]. Previous studies suggested that PDCoV infection can result in the formation of morphologically distinct multinuclear giant cells, known as syncytia [20, 30, 31]. The formation of syncytia may provide an additional route for the cell-to-cell transmission of PDCoV. It is not clear whether PDCoV uses cell–cell fusion for cell-to-cell transmission and, if so, what the underlying mechanisms and implications are.

In this study, using a cell–cell fusion system with cell-to-cell transmission assay, we demonstrated that PDCoV rapidly spreads via cell-to-cell transmission mediated by S-driven cell–cell fusion. We confirmed that PDCoV uses cell-to-cell transmission to resist neutralizing antibodies and immune sera. Our findings have revealed an unassessed mechanism of cell-to-cell transmission with the potential to impact PDCoV spread, pathogenesis, and antibody evasion.

## Materials and methods

### Cells, viruses, and reagents

HEK-293T, IPI-2I (porcine intestinal epithelial cells), ST (swine testis cells), Vero, and BHK-21 cells were cultured and maintained at 37°C and 5% CO_2_ in DMEM supplemented with 10% foetal bovine serum and 1% penicillin and streptomycin. Porcine aminopeptidase N (pAPN)-knockout IPI-2I (IPI-pAPN^KO^) cells and mouse monoclonal antibodies against PDCoV N or PDCoV S protein were prepared in our laboratory [32]. PDCoV strain CHN-HN-2014 (GenBank accession no. KT336560.1) was isolated from a piglet with acute diarrhoea in China in 2014 [10]. Rabbit monoclonal antibody against APN was purchased from Abcam (Cambridge, UK). Antibody against β-actin was purchased from ABclonal (Wuhan, China). Horseradish peroxidase-conjugated goat anti-mouse and horseradish peroxidase-conjugated goat anti-rabbit antibodies were purchased from Beyotime (Shanghai, China). Alexa Fluor 594-conjugated goat anti-mouse IgG was purchased from Abbkine (Wuhan, China). Polyclonal PDCoV-positive IgG was purified from hyperimmune sera using protein A + G affinity chromatography according to a standard protocol. Three anti-PDCoV-neutralizing monoclonal antibodies (NmAb-1, -2, -3), produced from hybridoma cells derived from Sp2/0 myeloma cells and spleen cells of BALB/c mice immunized with inactivated PDCoV virion, were gifted by Dr. Shusen Liao, Huazhong Agriculture University. The neutralizing titres (NT_50_), NmAb-1, NmAb-2, and NmAb-3, were 0.79, 1.12, and 0.7 μg/mL, respectively. Immune sera were raised in weaned piglets immunized with inactivated PDCoV vaccine. Inhibitors E64d, CA-074, Z-FY-CHO, Leupeptin, and Bafilomycin A1 (Baf-A1) were purchased from MCE (Shanghai, China). Pan-coronavirus fusion inhibitor EK1C4 was purchased from InvivoGen (San Diego, CA, USA).

### Plasmid construction

The codon-optimized PDCoV S gene was synthesized (Tsingke Biotech, Beijing, China) and cloned into the pUC57 vector with an EF-1α promoter to generate the pUC57-EF-1α-S expression plasmid. To enhance expression, woodchuck hepatitis virus posttranslational regulatory element and bovine growth hormone polyadenylation signal sequences were added downstream of the S gene. Expression constructs encoding pAPN, pCAGGS-Flag-pAPN were prepared as described previously [32]. pGL5-luc, pBind-ID, and PACT-Myod plasmids were gifts from Yandong Tang, Harbin Veterinary Research Institute, Chinese Academy of Agricultural Sciences.

### Indirect immunofluorescence assay

Treated cells were washed with phosphate-buffered saline three times, fixed with 4% paraformaldehyde, and permeabilized with methanol for 15 min at room temperature. The cells were blocked with 5% bovine serum albumin for 1 h, followed by incubation with a monoclonal antibody against the PDCoV N protein and Alexa Fluor 594-conjugated goat anti-mouse IgG antibody for 1 h. The cells were stained with DAPI to indicate the cell nuclei. Fluorescent images were taken at 10× magnification on an inverted fluorescence microscope (Olympus IX73, Japan).

### RNA extraction and RT-qPCR

To determine the cell-to-cell transmission and non-cell-to-cell infection of target cells by PDCoV, PDCoV-infected cells were collected, total RNA was extracted with TRIzol regent (Invitrogen, USA), and cDNA reverse transcribed with a reverse transcription kit (Vazyme, R223-01). RT-qPCR was performed with ChamQ Universal SYBR qPCR Master Mix (Vazyme, Q712-02) using primers N-F: 5′-AGCTGCTACCTCTCCGATTC-3′, N-R: 5′-ACATTGGCACCAGTACGAGA-3′.

### Cell-to-cell transmission assay

Uninfected target cells were seeded into 12-well plates and labelled with 5 μM 5-chloromethylfluorescein diacetate (CMFDA) (Yeasen, Shanghai, China) at 37°C for 30 min. IPI-2I or ST cells were inoculated with PDCoV (MOI = 1) in 2.5 μg/ml or 7.5 μg/ml trypsin at 37°C, respectively. At 12 h post-infection (hpi), PDCoV-infected (donor) cells were washed and trypsinized, then directly mixed with uninfected target cells (IPI-2I, ST, Vero, BHK-21, IPI-pAPN^KO^) growing in 12-well plates (cell-to-cell model) at ratio of 1:3 (donor: target). Simultaneously, an equivalent number of donor cells were seeded onto Transwell inserts (Corning, 0.4 μm pore size). Transwell inserts were suspended in wells already containing target cells, which prevented contact between donor and target cells but allowed the virus to pass through the Transwell insert membrane (non-cell-to-cell model). If needed, cells in the cell-to-cell model were overlaid with 1% methylcellulose during infection to prevent free virus infection. For experiments involving inhibitors, immune sera, or neutralizing antibodies, target cells were pre-treated with the indicated concentrations of reagents for 1 h and cocultured with donor cells in the presence of reagents throughout the infection process. After 24 h of coculture, indirect immunofluorescence assay (IFA), RT-qPCR, and 50% tissue culture infective dose (TCID_50_) assay were performed for detecting PDCoV replication in target cells.

Infection in the cell-to-cell model comprised both cell-to-cell transmission and non-cell-to-cell infection. The amount of cell-to-cell transmission was derived by subtracting the proportion of non-cell-to-cell infections performed in parallel. Non-cell-to-cell infections were evaluated by measuring the viral RNA copy numbers in target cells of the non-cell-to-cell model.

### Flow cytometry

Cocultured cells were harvested, washed twice, and analyzed by flow cytometry on a Bio-Rad S3e cell sorter (Bio-Rad, Hercules, CA, USA). The target cells labelled with CMFDA were sorted by fluorescence-activated cell sorting (FACS) based on green fluorescence, and subjected to RT-qPCR assay to detect viral RNA copy numbers.

### Cell–cell fusion assay

For the fluorescence-based cell–cell fusion assay, IPI-2I cells were inoculated with PDCoV (MOI = 1). After 12 h, PDCoV-infected cells were mixed with uninfected target cells, followed by coculture for 24 h. Cell–cell fusion was monitored by immunofluorescence staining using PDCoV N monoclonal antibody. Plates were photographed, and the nuclei in each syncytium were counted (three or more nuclei per multinucleated cell were considered a syncytium).

To quantify the cell–cell fusion, a luciferase-based cell–cell fusion assay was performed as previously described [30]. Briefly, donor cells seeded into six-well plates were co-transfected with 1 μg reporter plasmid pGL5-luc and 0.1 μg internal control plasmid pRL-TK with different doses of pCAGGS-Flag-pAPN. Target cells were co-transfected with 1 μg pBind-Id, 1 μg PACT-Myod, and the indicated amount of PDCoV S-expressing plasmid or empty vector. At 24 h post-transfection, donor and target cells were cocultured at a 1:1 ratio for 48 h. Cell–cell fusion activity was expressed as the relative activity of firefly luciferase to the activity of *Renilla* luciferase.

### Western blotting

Cells were lysed in lysis buffer (50 mM Tris-HCl, 150 mM NaCl, 1% NP-40, 10% glycerine, 0.1% SDS, 2 mM Na_2_EDTA, pH 7.4) for 30 min on ice, followed by centrifugation at 15,000 ×*g* for 20 min at 4°C. Cell lysate was boiled for 10 min with sample loading buffer (Beyotime). Samples were run on 7.5% SDS-PAGE gels and transferred to PVDF membranes, which were then incubated with primary antibody, followed by HRP-conjugated secondary antibody, and visualized using chemiluminescent substrate (Bio-Rad, CA, USA).

### Neutralization assay

Neutralizing virus titres were measured for serum samples that had been heat-inactivated at 56°C for 30 min. Virus titres were determined using a standard TCID_50_ assay. Briefly, 100 μl serum samples were serially diluted 2-fold with DMEM containing 7.5 μg/ml of trypsin. In each well, 50 μl diluted serum was mixed with 50 μl sample containing 100 TCID_50_ virus and incubated for 1 h at 37°C. Next, 100 μl virus/serum mixtures were added to wells containing LLC-PK1 cells. Control wells were exposed to the equivalent concentrations of control sera to ensure no cytopathic effect on uninfected cells or virus titre. After 3 days, the cytopathic effects (CPE) were observed and counted. The NT_50_ values of sera against PDCoV were calculated, which represented the lowest concentrations that protected >50% cells from CPE.

To quantify the neutralization of viruses either in cell-to-cell or non-cell-to-cell infections, uninfected IPI-2I cells were plated into a 12-well plate and allowed to rest for 1 h at 37°C in the presence of the increased concentration of NmAbs (1 × NT_50_ and 5 × NT_50_) or indicated concentration of immune serum (1 × NT_50_). Donor cells prepared by pre-infection of IPI-2I cells with PDCoV (MOI = 1) for 12 h were seeded into Transwell inserts (for non-cell-to-cell infection) or added directly to target cells on the bottom (for cell-to-cell infection). Cells were collected at 24 h post-coculture and subjected to RT-qPCR assay.

### Cell viability assay

Cell viability was measured using a cell counting kit-8 (CCK-8, Beyotime) in accordance with the manufacturer’s protocol.

### Statistical analysis

Data are shown as the mean ± standard deviation (SD). Statistical significance was determined using GraphPad Prism 6 software, and *P*-values were calculated using Student’s *t*-test and one-way ANOVA. Asterisks in the figures indicate the levels of significance (*, *P* < 0.05; **, *P* < 0.01; ***, *P* < 0.001).

## Results

### PDCoV efficiently spreads via cell-to-cell transmission in cell culture

**Figure 1.**
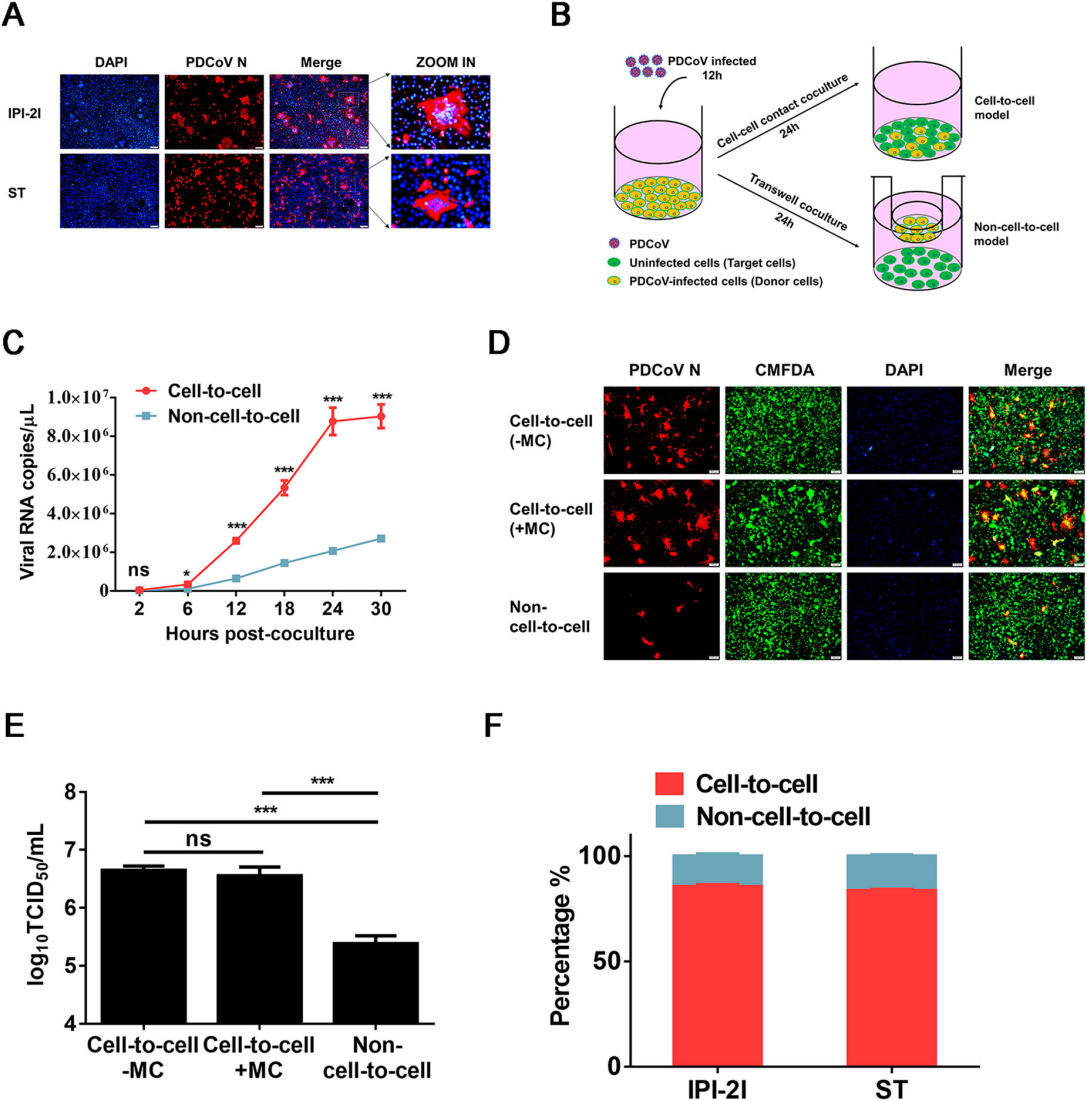
**PDCoV spreads via cell-to-cell transmission in cell culture**. (**A**) IPI-2I and ST cells were infected with PDCoV (MOI = 1) for 18 h. Cells were harvested and subjected to IFA with anti-PDCoV N antibody. Cell nuclei were stained with DAPI (blue). Scale bar, 100 μm. (**B**) Schematic representation of cell-to-cell and non-cell-to-cell infection assays (see details in Materials and Methods). (**C**) IPI-2I cells were infected with PDCoV (MOI = 1) for 12 h, collected, and added to target cells (cell-to-cell model) or Transwell inserts (non-cell-to-cell model). Cells were harvested for RT-qPCR assay at 2, 6, 12, 18, 24, 30 h post-coculture. (**D**, **E**) IPI-2I cells were infected with PDCoV (MOI = 1) for 12 h, then mixed with CMFDA-labelled target IPI-2I cells in the presence or absence of 1% methylcellulose or cultured separately with uninfected target cells by Transwell filters. After 24 h of coculture, cells were harvested and subjected to IFA (**D**) or TCID_50_ assay (**E**). (**F**) Cell-to-cell and non-cell-to-cell infection assays were performed on IPI-2I and ST cells as described in panel C. At 24 h post-coculture, ratios between cell-to-cell and non-cell-to-cell infection of PDCoV in IPI-2I and ST cells are displayed in stacked bars. Data represent means ± SD from three independent experiments. ***, *P* < 0.001; ns, not significant.

First, we assessed whether IPI-2I and ST cell lines form syncytia after PDCoV infection. As shown in Figure 1A, both IPI-2I and ST cell lines readily formed typical syncytia (cell–cell fusion) with multiple clustered nuclei upon PDCoV infection, whereas more and larger syncytia were formed in PDCoV-infected IPI-2I cells. To determine whether PDCoV uses cell–cell fusion for intracellular transmission, we used a cell–cell contact or Transwell coculture system to evaluate the cell-to-cell and non-cell-to-cell infection by PDCoV (Figure 1B and *Materials and Methods*) based on the viral RNA copy numbers in target cells. The results showed that cell-to-cell transmission of PDCoV was a much faster mode of transmission than non-cell-to-cell infection (Figure 1C). To further confirm that PDCoV spreads through cell-to-cell transmission, PDCoV-infected IPI-2I cells were mixed with CMFDA-labelled target cells, then the cocultured cells were overlaid with 1% methylcellulose to block free virus infection. The IFA results showed that, even if directly mixed cocultured cells were overlaid with methylcellulose, efficient syncytium formation was observed in the target cells (Figure 1D), suggesting that PDCoV spreads through cell-to-cell transmission. TCID_50_ assay results demonstrated no significant difference in viral titres in the cell-to-cell model with or without methylcellulose treatment, suggesting that PDCoV propagates efficiently in the absence of free virus infection (Figure 1E). Next, we compared cell-to-cell vs. non-cell-to-cell infection efficiencies in the coculture system at 24 h post-coculture. The results showed ∼80% cell-to-cell vs. ∼20% non-cell-to-cell infection by PDCoV in both IPI-2I and ST cells (Figure 1F). Taken together, these results reveal that PDCoV spreads rapidly and efficiently through cultured cells via cell-to-cell transmission.

### PDCoV infects non-susceptible cell lines through cell-to-cell transmission

**Figure 2.**
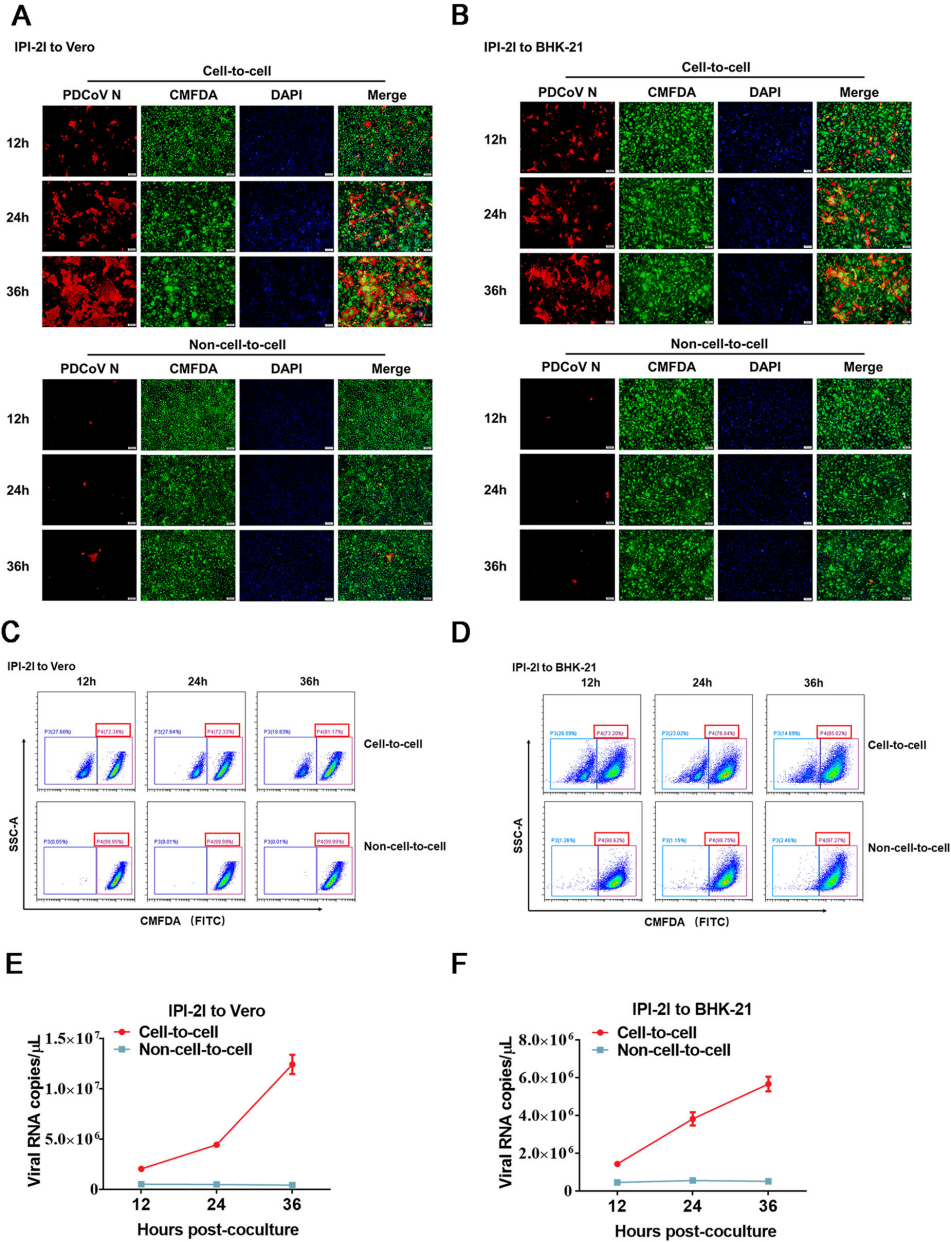
**PDCoV-producing IPI-2I cells transmit virus to non-susceptible cells through cell-to-cell transmission**. PDCoV-infected IPI-2I cells were cocultured with CMFDA-labelled Vero (**A**, **C**, **E**) and BHK-21 (**B**, **D**, **F**) cells. Cells were collected at 12, 24, 36 h post-coculture for IFA (**A, B**), flow cytometry (**C, D**), and RT-qPCR (**E, F**). (**A**, **B**) Cells were subjected to IFA with anti-PDCoV N antibody. Red arrows indicate syncytia formation in Vero (**A**) and BHK-21 (**B**) cells. Scale bar, 100 μm. (**C**, **D**) Cells were suspended in DMEM for sorting via flow cytometry. Flow cytometry dot plots show the percentage of Vero (**C**) and BHK-21 (**D**) cells in the cocultured cells. (**E**, **F**) RT-qPCR for detecting the viral RNA copy numbers in Vero (**E**) and BHK-21 (**F**) cells obtained by flow cytometry. Scale bar, 100 μm. Data represent means ± SD from three independent experiments.

To determine whether PDCoV can infect non-susceptible cells via cell-to-cell transmission, BHK-21 cells or Vero cells, which are poorly susceptible to PDCoV infection, were cocultured with PDCoV-infected IPI-2I cells. In a cell-to-cell model, we observed a gradually increased numbers of PDCoV positive cells co-stained with CMFDA-labelled Vero and BHK-21 cells 12 h after the initiation of coculture (Figure 2A and B), indicating the transmission of PDCoV to the target cells. In contrast, few infected cells were detected in the non-cell-to-cell model (Figure 2A and B). To further confirm the result, we performed flow sorting to collect CMFDA-labelled Vero or BHK-21 cells and detected the viral copy numbers in these cells under the same condition described in Figure 2A and B. As expected, the results of flow cytometric analysis showed that Vero or BHK-21 cells accounted for >70% of the cocultured cells in the cell-to-cell model (Figure 2C and D). RT-qPCR assay showed a rapid increase in viral RNA copy numbers for cell-to-cell transmission but not non-cell-to-cell infection, suggesting that PDCoV spread efficiently in the Vero or BHK-21 cells by cell-to-cell transmission rather than non-cell-to-cell infection (Figure 2E and F). Taken together, these results suggest that cell-to-cell transmission plays a critical role in PDCoV infection of non-susceptible cells.

### PDCoV S-mediated cell–cell fusion contributes to viral cell-to-cell transmission

**Figure 3.**
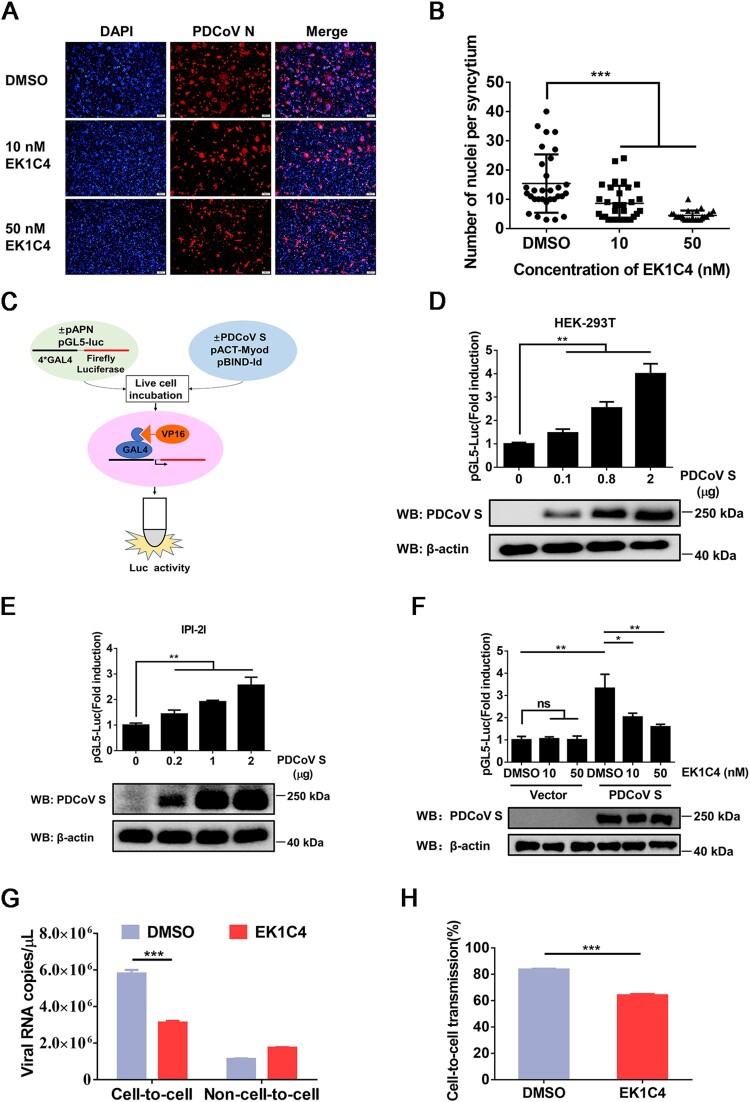
**Cell–cell fusion mediated by PDCoV S protein contributes to cell-to-cell transmission**. (**A**) PDCoV-infected IPI-2I cells were mixed with uninfected IPI-2I cells and cocultured in the presence of indicated concentrations of EK1C4 for 24 h, followed by IFA. Scale bar, 100 μm. (**B**) Numbers of nuclei per syncytium in panel A are displayed in the scatter plot. (**C**) Schematic diagram of luciferase-based cell–cell fusion assay (see details in Materials and Methods). (**D**, **E**) HEK-293T (**D**) and IPI-2I (**E**) cells were co-transfected with pBind-Id, PACT-Myod, and increasing quantities of pUC57-EF-1α-S expression plasmids or empty vector and cocultured with pGl5-luc-expressing cells for 48 h, followed by dual-luciferase assay. The expression of S protein and β-actin was detected via western blotting assay with antibodies against PDCoV-S and β-actin, respectively. β-actin served as a protein loading control. (**F**) HEK-293T cells were co-transfected with pUC57-EF-1α-S, pBind-Id, and PACT-Myod and mixed with other HEK-293T cells co-transfected with pGl5-Luc and pRL-TK. Cells were cocultured in fresh media containing 10 nM or 50 nM EK1C4 or DMSO. After 48 h of coculture, cell–cell fusion was evaluated by dual-luciferase assay. Expression of S protein and β-actin was detected via western blotting. (**G**) PDCoV-infected IPI-2I cells were cocultured with uninfected IPI-2I cells with 50 nM EK1C4 or DMSO for 24 h, followed by RT-qPCR assay. (**H**) Ratio of cell-to-cell transmission to total infection is calculated in panel G. Data represent means ± SD from three independent experiments. *, *P* < 0.05; **, *P* < 0.01; ***, *P* < 0.001; ns, not significant.

Cell–cell fusion is considered an important mechanism of cell-to-cell transmission for a number of enveloped viruses [27]. To explore whether cell–cell fusion plays a role in the cell-to-cell transmission of PDCoV, EK1C4, a pan-CoV fusion inhibitor with potent inhibitory activity against divergent CoVs [33], was applied to cell–cell fusion and cell-to-cell transmission assays. We first performed the cell viability assay for EK1C4 in IPI-2I cells using a cell counting kit-8 and found that no obvious cytotoxicity could be observed when the concentration of EK1C4 was up to 50 nM (data not shown). However, EK1C4 significantly inhibited the syncytium formation of IPI-2I cells after PDCoV infection (Figure 3A and B). We performed a luciferase-based cell–cell fusion assay to determine the effect of EK1C4 on cell–cell fusion mediated by the S protein (Figure 3C). The results showed that ectopic expression of the PDCoV S protein significantly increased cell–cell fusion in a dose-dependent manner in both HEK-293T and IPI-2I cells (Figure 3D and E); however, the increase was effectively inhibited by EK1C4 in a dose-dependent manner (Figure 3F). We next explored whether cell–cell fusion mediated by PDCoV S plays a crucial role in cell-to-cell transmission. As shown in Figure 3G and H, EK1C4 treatment significantly inhibited the cell-to-cell transmission, but not non-cell-to-cell infection, of PDCoV, leading to a lower ratio of cell-to-cell transmission to total infection. Altogether, these results indicate that PDCoV S-mediated cell–cell fusion contributes to efficient cell-to-cell transmission.

### pAPN enhances cell-to-cell transmission of PDCoV

**Figure 4.**
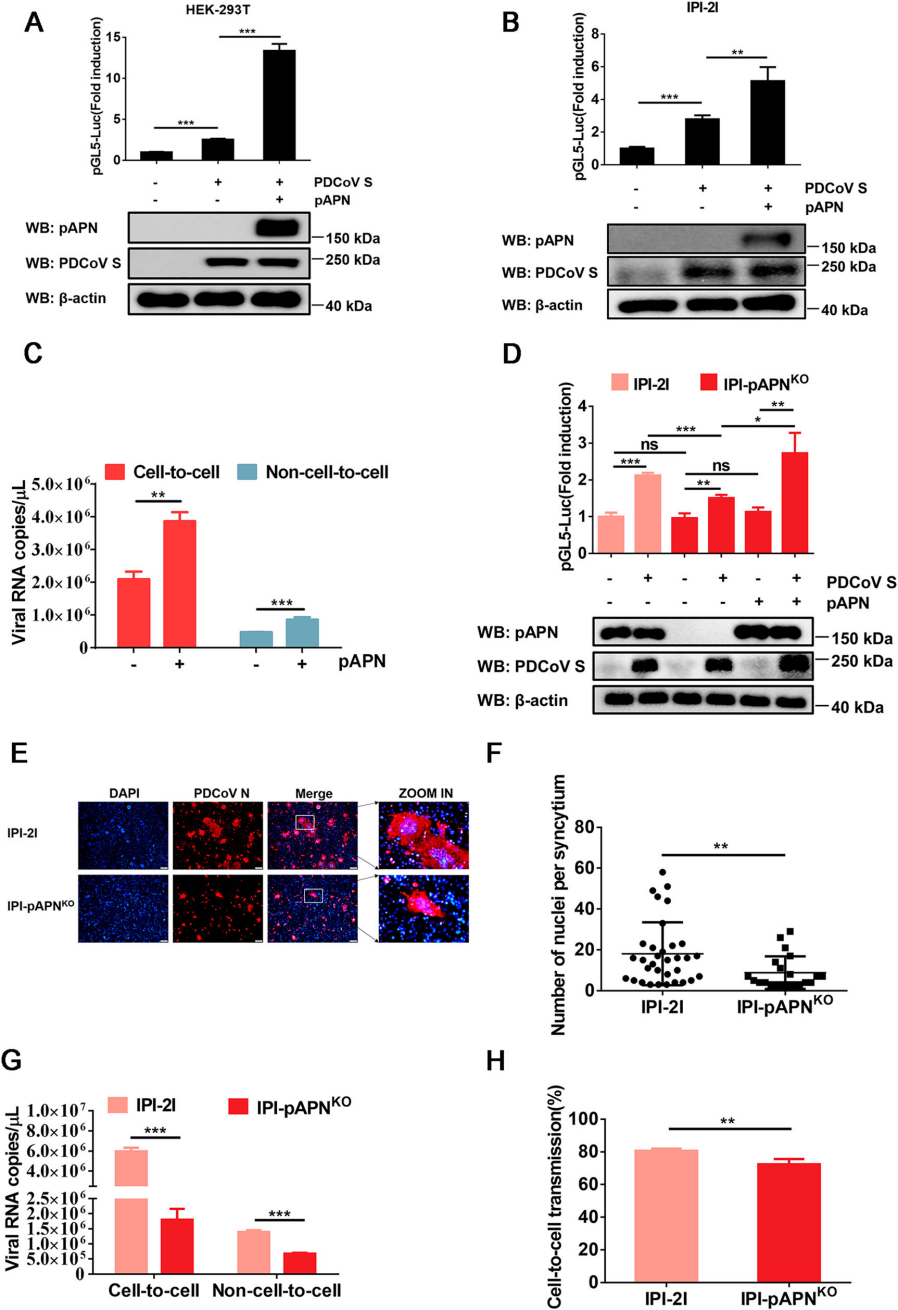
**pAPN enhances cell-to-cell transmission of PDCoV**. (**A**, **B**) HEK-293T (**A**) and IPI-2I cells (**B**) were co-transfected with pBind-Id, PACT-Myod, and 1 µg of pUC57-EF-1α-S or empty vector and cocultured with pGl5-luc-expressing cells transfected with 1 µg of pCAGGS-Flag-pAPN or pCAGGS-Flag. Dual-luciferase assay was performed at 48 h after coculture. The expression of pAPN, PDCoV S, and β-actin was verified via western blotting. (**C**) IPI-2I cells were transfected with 1 µg of pCAGGS-Flag-pAPN or pCAGGS-Flag. At 24 h post-transfection, transfected cells were mixed with PDCoV-infected IPI-2I cells, followed by coculture for 24 h. Cells were harvested for RT-qPCR assay. (**D**) IPI-2I or IPI-pAPN^KO^ cells were transfected with indicated plasmid and cocultured for 48 h. Cell–cell fusion was evaluated by dual-luciferase assay. Expression of endogenous pAPN and PDCoV was detected by western blotting. (**E**) PDCoV-infected IPI-2I cells were cocultured with uninfected IPI-2I or IPI-pAPN^KO^ cells for 24 h, followed by IFA for detecting syncytium formation. Scale bar, 100 μm. (**F**) Numbers of nuclei per syncytium in panel E are displayed in the scatter plot. (**G**) Uninfected IPI-pAPN^KO^ cells or IPI-2I cells were mixed with PDCoV-infected IPI-2I cells, cocultured for 24 h, and subjected to RT-qPCR assay. (**H**) Ratio of cell-to-cell transmission to total infection is calculated in panel G. Data represent means ± SD from three independent experiments. *, *P* < 0.05; **, *P* < 0.01; ***, *P* < 0.001; ns, not significant.

pAPN is a functional receptor of PDCoV that mediates viral entry to host cells [34, 35]. We evaluated the effect of pAPN on the cell-to-cell transmission of PDCoV and found that ectopic expression of pAPN enhanced the cell–cell fusion mediated by PDCoV S (Figure 4A and B) as well as cell-to-cell and non-cell-to-cell infection (Figure 4C). To further determine whether pAPN is critical to the cell-to-cell transmission of PDCoV, IPI-pAPN^KO^ cell lines were used in subsequent experiments. As revealed by a luciferase-based cell–cell fusion assay, APN ablation in IPI-2I cells reduced PDCoV S-mediated cell–cell fusion by ∼30% compared with that seen in parental IPI-2I cells, whereas overexpression of pAPN in IPI-pAPN^KO^ cells restored the cell–cell fusion mediated by PDCoV S to levels comparable to those observed in parental IPI-2I cells (Figure 4D), confirming that cell–cell fusion mediated by PDCoV S is regulated by pAPN. We also compared the syncytium formation of parental IPI-2I cells and IPI-pAPN^KO^ cells upon PDCoV infection and found that the cell–cell fusion induced by PDCoV was significantly reduced in the absence of pAPN (Figure 4E and F). In accordance with the effect of pAPN on cell–cell fusion, pAPN ablation in IPI-2I cells resulted in reduced cell-to-cell transmission and lowered the rate of virus cell-to-cell transmission to total infection (Figure 4G and H). Collectively, these results demonstrate that pAPN plays a positive role in the cell-to-cell transmission of PDCoV.

### Endosomal pathway is involved in cell-to-cell transmission of PDCoV

**Figure 5.**
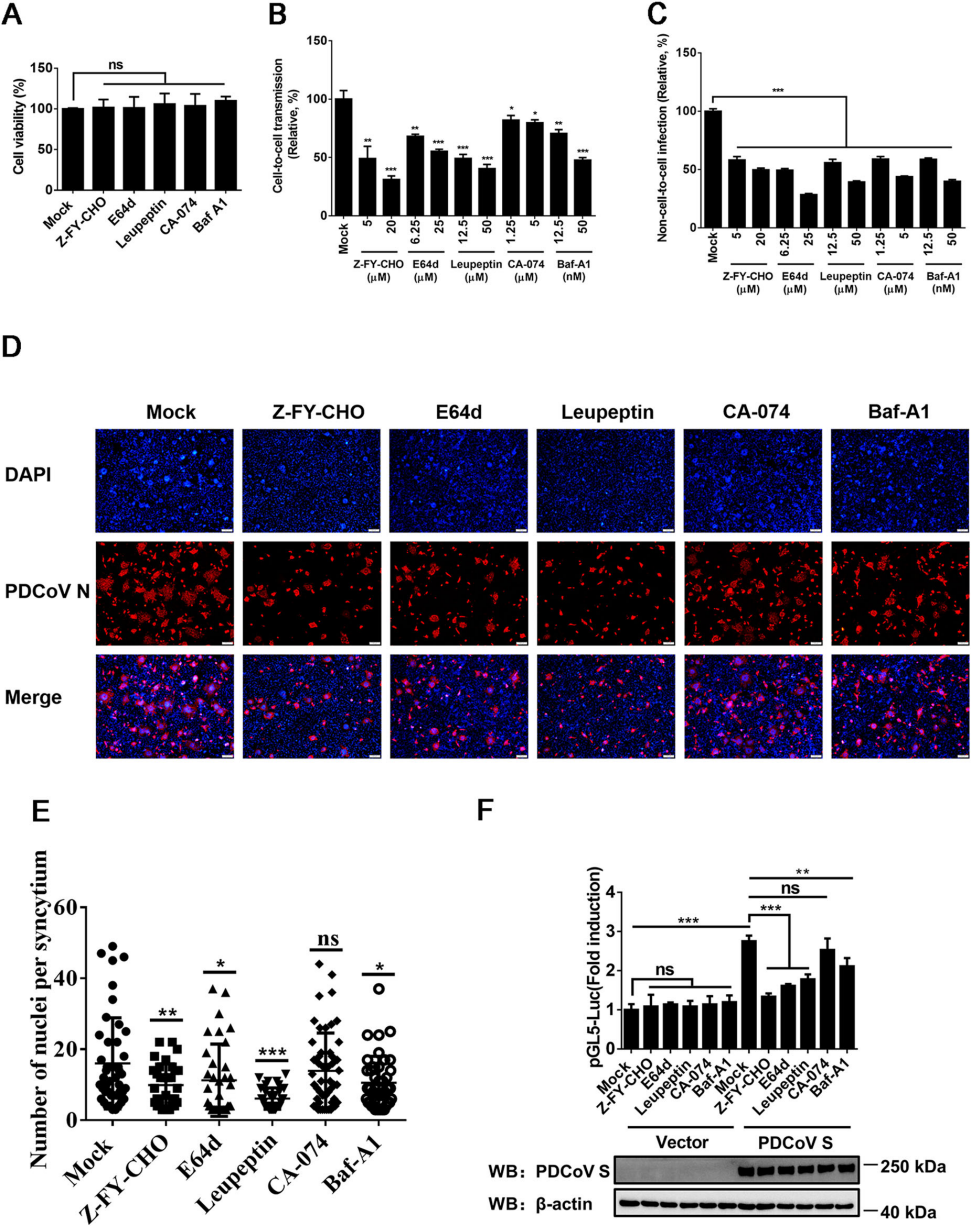
**Cathepsins in endosomes are involved in cell-to-cell transmission of PDCoV**. (**A**) IPI-2I cells cultured in 96-well plates were incubated with leupeptin (50 µM), Baf-A1 (50 nM), E64d (25 µM), Z-FY-CHO (20 µM), or CA-074 (5 µM). After 24 h incubation, 10 μl of CCK-8 was added to the cells and incubated for 1 h at 37°C, followed by measurement of OD value at 450 nm. (**B**, **C**) Cell-to-cell (**B**) and non-cell-to-cell **(C)** infection assays were performed on IPI-2I cells as described in the legend of Figure 1, except that indicated inhibitors were present during the infection period. At 24 h post-coculture, cells were harvested and subjected to RT-qPCR assay. Ratios of relative infection were plotted by setting mock group values to 100. (**D**) PDCoV-infected IPI-2I cells were mixed with uninfected IPI-2I and cocultured in the presence of indicated concentration of inhibitors used in panel A for 24 h, followed by IFA for detecting syncytium formation. Scale bar, 100 μm. (**E**) Nuclei in each syncytium in panel D were counted. (**F**) Luciferase-based cell–cell fusion assays were performed on HEK-293T cells as described in the legend of Figure 3D, except that indicated concentration of inhibitors used in panel A were included during coculture. Data represent means ± SD from three independent experiments. *, *P* < 0.05; **, *P* < 0.01; ***, *P* < 0.001; ns, not significant.

PDCoV uses two pathways for entry that are either protease-mediated at the plasma membrane and/or cathepsins in the endosome [20]. To evaluate the effects of the endosomal pathway on cell-to-cell and non-cell-to-cell infection with PDCoV, a panel of endosomal protease inhibitors, including the pan-spectrum protease inhibitor leupeptin, the lysosomal acidification inhibitor Baf-A1, the pan-cysteine cathepsin inhibitor E64d, the cathepsin L-specific inhibitor Z-FY-CHO, and the cathepsin B-specific inhibitor CA-074, were used in parallel. A CCK-8-based cell viability assay showed no cytotoxicity in cells treated with these inhibitors at the concentrations used in our experiments (Figure 5A). As shown in Figure 5B and C, all endosomal protease inhibitors used significantly inhibited cell-to-cell transmission and non-cell-to-cell infection by PDCoV in a dose-dependent manner. These results support the notion that the endosomal pathway is involved in the cell-to-cell transmission of PDCoV, but it appears to play a less dominant role compared with non-cell-to-cell infection.

To further investigate whether an endosomal pathway is associated with PDCoV S-mediated cell–cell fusion, the inhibitors were applied in cell–cell fusion assays. The results showed that leupeptin, Baf-A1, E64d, and Z-FY-CHO significantly reduced the cell–cell fusion induced by PDCoV (Figure 5D and E). A luciferase-based cell–cell fusion assay showed that, except for CA-074, all inhibitors markedly inhibited PDCoV S-driven cell–cell fusion (Figure 5F). CA-074 had modest effects on cell-to-cell transmission of PDCoV and PDCoV S-mediated cell–cell fusion, indicating that cathepsin B may be less important than cathepsin L in the endosomal pathway used by PDCoV. These results suggest that an endosomal pathway is involved in the cell-to-cell transmission of PDCoV.

### Cell-to-cell transmission of PDCoV is relatively refractory to neutralizing antibody

**Figure 6.**
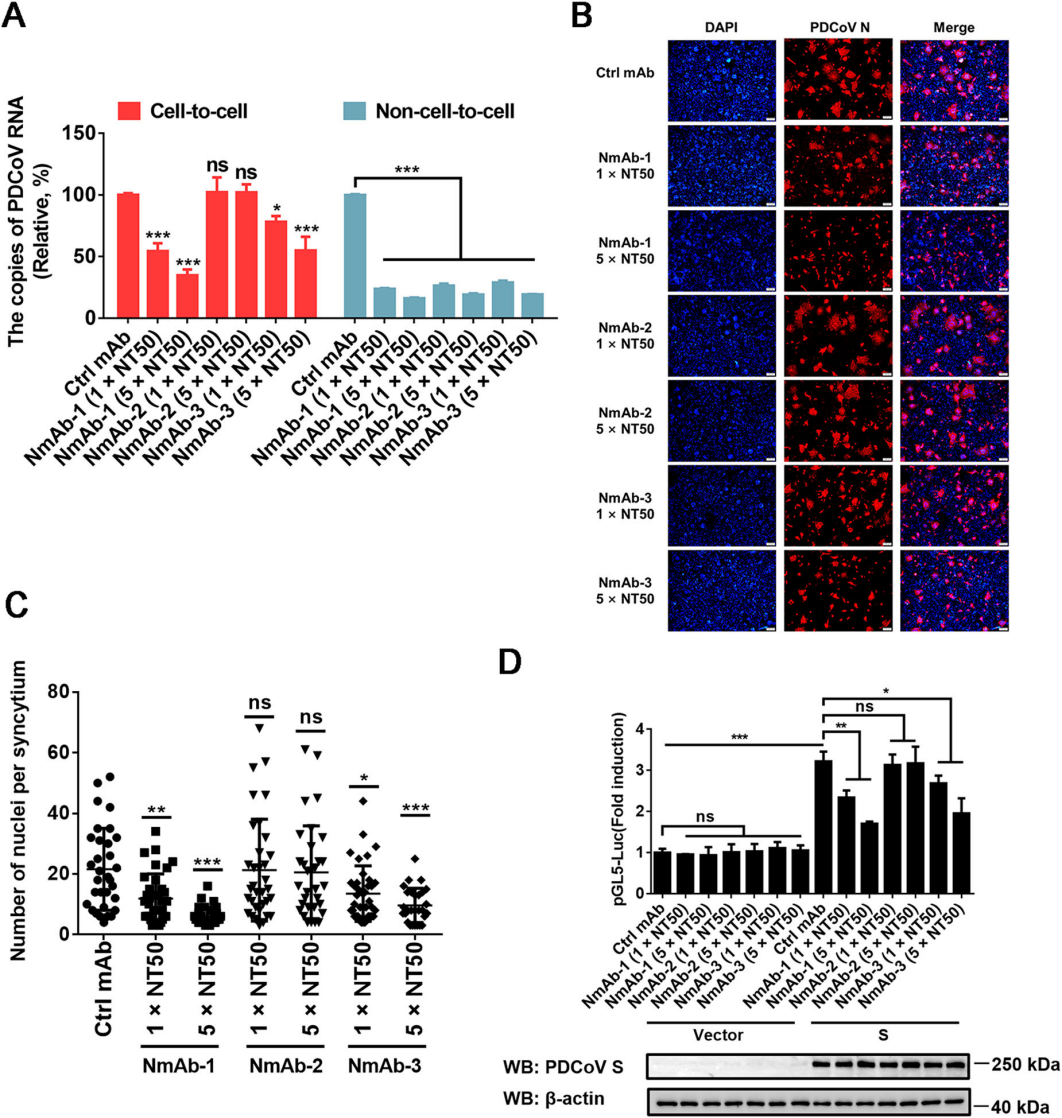
**Cell-to-cell transmission of PDCoV is refractory to NmAbs**. (**A**) Cell-to-cell and non-cell-to-cell assays were carried out on IPI-2I cells as described in the legend of Figure 1, except indicated concentrations of NmAb-1, -2, or -3 were included during the infection period. Relative viral RNA copy numbers were plotted by setting the values of the control monoclonal antibody group to 100% for statistical analyzes. (**B**) PDCoV-infected cells were mixed with uninfected IPI-2I cells and cocultured in the presence of indicated concentration of NmAbs. Photos of syncytia formation were taken at 24 h after coculture and presented. (**C**) Numbers of nuclei per syncytium in panel B were determined and displayed in the scatter plot. (**D**) Luciferase-based cell–cell fusion assays were performed in HEK-293T cells as described in the legend of Figure 3D, except that indicated concentration of NmAbs were included during coculture. Data represent means ± SD from three independent experiments. *, *P* < 0.05; **, *P* < 0.01; ***, *P* < 0.001; ns, not significant.

To examine the sensitivity of cell-to-cell transmission of PDCoV to S-specific neutralizing monoclonal antibodies (NmAbs), three NmAbs, NmAb-1, NmAb-2 and NmAb-3, were used. RT-qPCR results showed that all three NmAbs effectively inhibited the non-cell-to-cell infection of PDCoV, but only NmAb-1 and NmAb-3 significantly suppressed cell-to-cell transmission of PDCoV and the inhibitory effects were in a dose-dependent manner (Figure 6A). We also performed cell–cell fusion assays to explore the effects of these NmAbs on cell–cell fusion. Interestingly, the results indicated that PDCoV S-mediated cell–cell fusion was inhibited by NmAb-1 and NmAb-3 in a dose-dependent manner (Figure 6B-D). In contrast, NmAb-2 had no significant effect on cell–cell fusion, which is consistent with the observation that NmAb-2 failed to impede cell-to-cell transmission of PDCoV but effectively prevented free virus infection. These results revealed that the cell-to-cell transmission of PDCoV is relatively refractory to neutralization by NmAbs.

### PDCoV cell-to-cell spread is insensitive to immune sera

**Figure 7.**
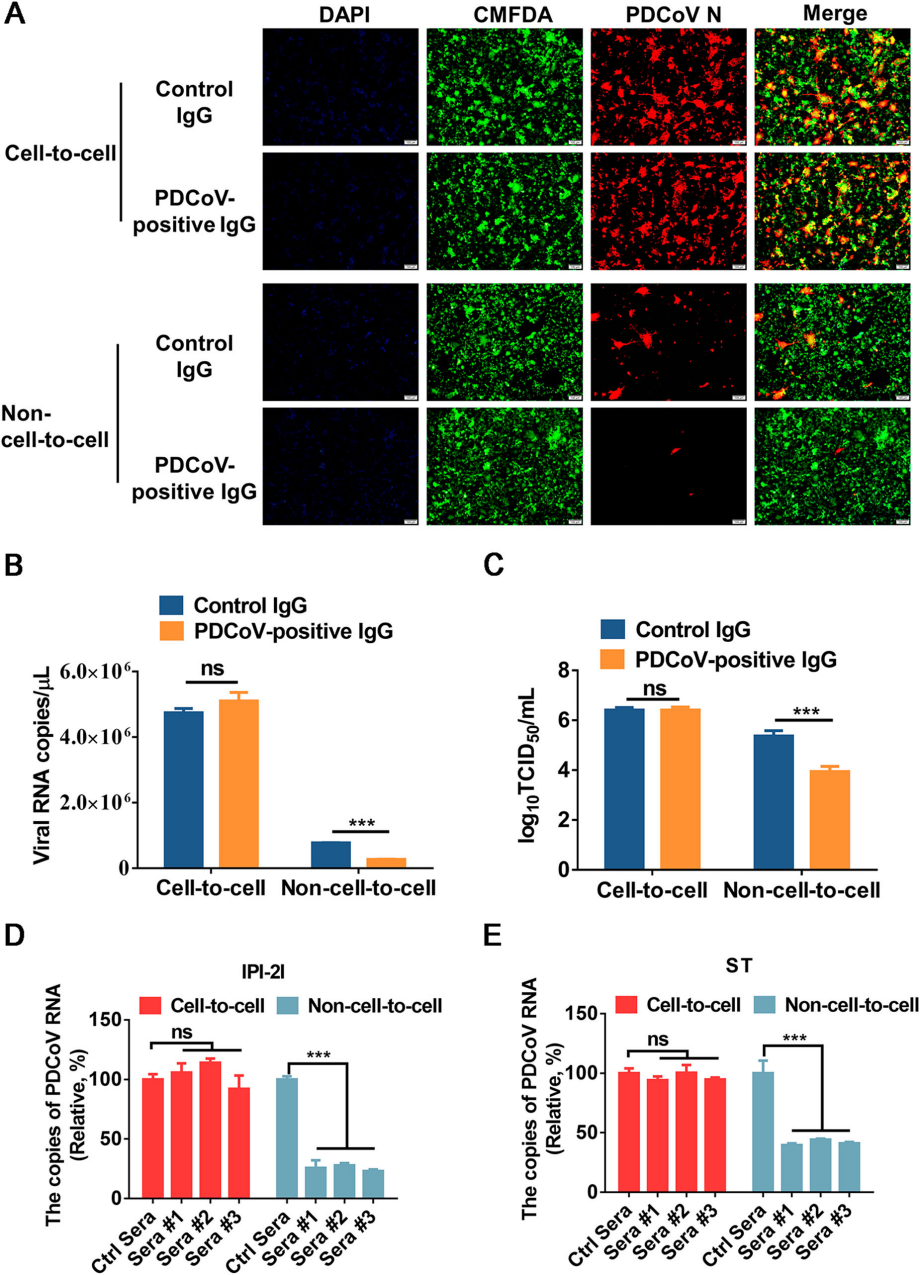
**Cell-to-cell transmission of PDCoV is resistant to immune sera**. (**A-C**) PDCoV-infected IPI-2I cells were mixed with uninfected IPI-2I cells and cocultured in the presence of PDCoV-positive IgG or control IgG for 24 h. The cells were collected and subjected to IFA (**A**), RT-qPCR (**B**), or TCID_50_ assay (**C**). (**D**, **E**) PDCoV-infected IPI-2I (**D**) and ST (**E**) cells were cocultured with IPI-2I and ST cells separately in the presence or the absence of immune sera for 24 h. Relative viral RNA copy numbers were plotted by setting the values of control sera group to 100% for statistical analyzes. ***, *P* < 0.001. ns, not significant.

We further tested the sensitivity of cell-to-cell transmission and non-cell-to-cell infection of PDCoV to neutralization by immune sera. To avoid possible interference from serum components, the IgG fractions of porcine polyclonal antibodies were purified from hyperimmune sera raised against PDCoV. The purified IgG was added to the cocultured medium for 24 h, and viral spread via cell-to-cell transmission and non-cell-to-cell infection was analyzed by IFA, RT-qPCR, and TCID_50_ assays. The results showed significantly reduced viral RNA copy numbers and viral titres in non-cell-to-cell infection in the presence of PDCoV-positive IgG in comparison to the control IgG group (Figure 7A-C). In contrast, the potently neutralizing PDCoV-positive IgG had no significant inhibitory effect on syncytium formation, viral RNA copy number, or viral titre in cell-to-cell transmission (Figure 7A-C), suggesting that cell-to-cell transmission of PDCoV is resistant to the PDCoV-positive IgG that potently neutralizes free viruses. We also tested three sera from three individuals immunized with an inactivated PDCoV vaccine and observed that they failed to significantly inhibit cell-to-cell transmission in both IPI-2I and ST cells, despite their capacity to efficiently block free virus infection (Figure 7D and E). Collectively, these results indicated that the cell-to-cell transmission of PDCoV is insensitive to immune sera.

## Discussion

Viral cell-to-cell transmission has received more attentions in recent years because it may be a common route used by many highly pathogenic viruses to spread rapidly and efficiently [21–23, 36, 37]. Compared with the cell-free route, viral cell-to-cell transmission is thought to be a much more efficient mechanism for spread, allowing viral evasion of the effects of neutralizing antibodies and other immune system components and leading to therapy failure/resistance and maintenance of infection [38–40]. Despite cell-to-cell transmission being important for viral spread, the cell biology and molecular players involved in this mechanism are poorly understood. In this work, we confirmed that PDCoV can efficiently spread through cell-to-cell transmission mediated by PDCoV S-induced cell–cell fusion, and that pAPN and cathepsins are involved in the cell-to-cell transmission of PDCoV. Importantly, the cell-to-cell transmission of PDCoV was, to a large extent, resistant to antibody neutralization.

Many enveloped viruses use cell–cell fusion processes and syncytium formation in cell-to-cell transmission [27]. In a recent study, Yang et al. showed that PDCoV induced cell–cell fusion in LLC-PK1 cells, but not ST cells, and they found that PDCoV spread in LLC-PK1 cells very efficiently via cell-to-cell transmission [30]. However, there was a lack of strong evidence supporting a direct correlation between cell–cell fusion and cell-to-cell transmission, and the detailed mechanisms and biological significances were not further explored. In this study, using IPI-2I cells, a line of intestinal epithelial cells more physiologically relevant to PDCoV infection *in vivo*, we provided evidence to support the conclusion that PDCoV uses S-mediated cell–cell fusion as an alternative pathway for cell-to-cell transmission, as demonstrated by the pan-coronavirus fusion inhibitor EK1C4, pAPN-knockout, and endosomal protease inhibitors significantly attenuating PDCoV S-mediated cell–cell fusion and viral cell-to-cell transmission (Figures 3–5). Our results showed that both IPI-2I and ST cell lines formed efficient syncytia upon PDCoV infection, whereas more and larger syncytia were formed in PDCoV-infected IPI-2I cells. Another study showed that PDCoV infection induces syncytia formation in ST cells but not IPI-2I cells [20]. We speculated that IPI-2I and ST cell lines of different lineages and generations display differences in their abundance of receptor(s), proteases, and other unidentified host factors that regulate PDCoV-induced cell–cell fusion. The detailed mechanisms contributing to these differences remain to be further investigated. Interestingly, we found that PDCoV induced extensive syncytial formation in non-susceptible cells when PDCoV-infected IPI-2I cells were cocultured with non-susceptible cells. This prompted us to propose that, once infection is established, PDCoV rapidly spread intracellularly in a receptor-independent manner by inducing cell–cell fusion. However, more experiments need to be performed to confirm this hypothesis. Overall, we demonstrated that cell–cell fusion plays an important role in transmitting PDCoV to non-susceptible cells in our experimental conditions.

Cell–cell fusion is mainly mediated by specific interactions between certain viral fusion proteins and surface molecules or receptors expressed on neighbouring non-infected cells [41], such as dipeptidyl peptidase 4 (DPP4) and angiotensin-converting enzyme 2 (ACE2) used by Middle East respiratory syndrome coronavirus (MERS-CoV) and SARS-CoV-2, respectively [42, 43]. The receptor(s) of PDCoV has not been clearly elucidated. Previous studies showed that APN binds to the receptor-binding domain of the PDCoV S protein and is responsible for S-mediated viral entry [34, 44]. A recent study indicated that pAPN mediates PDCoV entry by an endocytotic pathway to establish efficient viral replication [35]. However, in *in vitro* and *in vivo* studies, pAPN-knockout failed to completely block PDCoV infection, indicating APN is not a key receptor for PDCoV [32, 45, 46]. In this study, we found that APN enhances the cell–cell fusion mediated by PDCoV S and PDCoV cell-to-cell transmission, but it is not strictly required for these processes (Figure 4), which is consistent with previous studies. In addition, BHK-21 and Vero cells that lack APN expression still exhibited giant syncytia formation after they were mixed with PDCoV-infected IPI-2I (Figure 2). Obvious cell-to-cell transmission was detected, whereas little free virus infection was observed in BHK-21 and Vero cells. Taken together, the evidence suggests APN is not an absolute requirement for PDCoV infection, but the presence of APN greatly increases both cell–cell fusion mediated by S protein and cell-to-cell transmission of PDCoV. Our results showed that endosomal cathepsins had a positive role in S-driven cell–cell fusion and cell-to-cell transmission, especially cathepsin L, which played a dominant role in these processes (Figure 5). These results are reminiscent of those of prior studies of other coronaviruses, including SARS-CoV, MERS-CoV, and SARS-CoV-2, in which cathepsin L was shown to play a more important role than cathepsin B in cleaving the S protein to drive fusion [47–49]. It is of note that, although CA-074 had no obvious effect on cell–cell fusion, it still exhibited a certain inhibitory effect on the cell-to-cell transmission of PDCoV, suggesting that, in addition to cell–cell fusion, an endosomal pathway may also regulate cell-to-cell transmission by other unknown mechanisms. Further exploration of the exact cellular cofactors involved in the cell-to-cell transmission of PDCoV should deepen our understanding of this viral pathogenesis and contribute to uncovering new therapeutic approaches, either in terms of novel viral targets required for viral cell-to-cell transmission or cellular targets that facilitate this mode of virus spread.

One purpose of cell-to-cell transmission is to protect viruses from the effects of antibody neutralization. In this study, we found that neither the NmAbs nor immune sera used in this study inhibited cell-to-cell transmission to a large extent, despite their capacity to almost completely block non-cell-to-cell infection by PDCoV (Figure 6 and 7). However, few antibodies have been probed for their effect on cell-to-cell transmission. Interestingly, our results showed that three NmAbs significantly inhibited free virus infection; however, they displayed different degrees of inhibitory effects on cell–cell transmission. Of the three, NmAb-2 did not suppress the cell-to-cell transmission of PDCoV; while NmAb-1 and NmAb-3 exhibited obvious inhibitor effects on the cell-to-cell transmission of PDCoV, but NmAb-1 possessed the greatest inhibitory effect. The effects of three NmAbs on cell-to-cell transmission are completely consistent with their inhibitory effects on cell–cell fusion (Figure 6), further supporting the notion that PDCoV S-mediated cell–cell fusion contributes to the cell-to-cell transmission of this virus. It should be noted that the three NmAbs used in this study have been demonstrated to target the conformational epitopes of PDCoV S protein. The action mechanisms reported for virus-neutralizing antibodies include (1) through competitive inhibition of receptor binding for blocking attachment; (2) denaturation of native viral fusion protein conformations [50], and (3) pre-fusion trapping [51] for blocking fusion. For the neutralizing antibodies against coronavirus S protein, Yao et al. reported that the neutralizing antibody P17 is able to block SARS-CoV-2 S-induced cell–cell fusion by blocking the binding of SARS-CoV-2 RBD to its ACE2 receptor and restraining the conformational changes of spike [52]; Asarnow et al. demonstrated that the neutralizing antibody 5A6 suppresses SARS-CoV-2 spike-mediated cell–cell fusion through a synergistic effect between receptor blockade and pre-fusion trapping [53]. Thus, we speculated that the two NmAbs (NmAb-1 and NmAb-3) may employ similar or other unknown mechanisms to suppress cell–cell fusion which in turn, inhibits the cell-to-cell transmission of PDCoV. These findings suggest that the neutralization of free viruses is insufficient to block PDCoV infection; therefore, approaches that suppress viral infection via cell-to-cell transmission should also be considered. Further studies are needed to elucidate the extent to which cell-to-cell transmission contributes to PDCoV spread *in vivo* and whether this process is involved in viral pathogenesis and confers the ability to evade neutralizing antibodies upon the virus. If this mechanism is indeed central to PDCoV infection and pathogenesis, then the development of therapeutic vaccines, neutralizing antibodies, and inhibitors that directly block this process is critical to preventing the spread of this viral infection.

In summary, we propose a hypothetical model to illustrate the roles of cell-to-cell transmission in the spread of PDCoV. On infecting cells, PDCoV mainly uses cell–cell fusion to self-transmit to neighbouring non-infected cells, rather than diffusing through the extracellular environment and infecting new cells. The functional receptor pAPN and cathepsins in endosomes are involved in this mode of virus spread. More importantly, the cell-to-cell transmission of PDCoV exhibits resistance to antibody neutralization. These findings expand our knowledge of the spread of PDCoV infection and deepen our understanding of the pathogenic and humoral immunity mechanisms of PDCoV.

## Data Availability

The data that support for the findings of this study are all contained in the manuscript.
